# Employment-Related Assistive Technology Needs in Autistic Adults: A Mixed-Methods Study

**DOI:** 10.3390/ejihpe15090170

**Published:** 2025-08-26

**Authors:** Kaiqi Zhou, Constance Richard, Yusen Zhai, Dan Li, Hannah Fry

**Affiliations:** 1 Department of Rehabilitation and Health Services, University of North Texas, Denton, TX 76203, USA; 2 Wisconsin Center for Education Research, University of Wisconsin-Madison, Madison, WI 53706, USA; carichard3@wisc.edu (C.R.); hfry@wisc.edu (H.F.); 3 Department of Human Studies, University of Alabama at Birmingham, Birmingham, AL 35233, USA; yzhai@uab.edu; 4 School of Human Development and Organizational Studies in Education, University of Florida, Gainesville, FL 32601, USA; 5 Department of Health Promotion Sciences, University of Oklahoma Health Sciences, Oklahoma City, OK 73104, USA; dan-li@ou.edu

**Keywords:** autism, assistive technology, employment, needs assessment, workplace accommodations

## Abstract

**Background:** Assistive technology (AT) can support autistic adults in navigating employment-related challenges. However, limited research has explored autistic adults’ actual needs and experiences with AT in the workplace. Existing studies often overlook how well current AT solutions align with the real-world demands autistic adults face across the employment process. To address this gap, this study conducted a needs assessment to explore autistic adults’ perceived AT and AT service needs across employment stages, identify satisfaction and discontinuation patterns, and examine barriers and facilitators to effective use. **Methods:** A total of 501 autistic adults were recruited through an online crowdsourcing platform, Prolific. Participants completed a needs assessment that included Likert-scale items and open-ended questions. Quantitative data were analyzed using descriptive statistics and weighted needs scoring procedures. Thematic analysis was applied to qualitative responses regarding satisfaction, discontinuation, and general reflections on AT use. **Results:** Job retention received the highest total weighted needs score, followed closely by skill development and job performance. Participants reported lower perceived needs for AT in the job development and placement domain. Qualitative findings revealed that AT was described as essential for daily functioning and independence, but barriers such as limited access, inadequate training, and social stigma affected use. Participants also emphasized the need for more person-centered and context-specific AT services. **Conclusions:** AT has the potential to significantly enhance employment outcomes for autistic adults. However, current services often lack personalization and alignment with real-world needs. Findings support the development of more inclusive, tailored, and accessible AT solutions across all employment stages.

## 1. Introduction

People’s understanding of autism has been evolving throughout history. From a clinical standpoint, autism spectrum disorder (ASD) refers to a neurodevelopmental condition characterized by persistent social communication difficulties and restricted, repetitive behaviors, interests, or activities (American Psychiatric Association, 2022). However, the autistic community strongly criticizes this pathological framework, arguing that being autistic should not be defined solely by deficits but instead recognized as a valuable aspect of human diversity and an inseparable part of who they are (den Houting, 2019).

Regardless of how autism is understood, its prevalence is undergoing a significant increase both globally and in the United States (Maenner et al., 2023; Zeidan et al., 2022). Being autistic is a unique experience, with highly variable individual differences (Anderson-Chavarria, 2022). Each autistic individual experiences different levels of challenges in areas including, but not limited to, social communication, sensory processing, information processing, executive functioning, emotional and stress regulation, and motor coordination (Buch et al., 2023; Di Vara et al., 2024). Autism is also described as a psychosocial disorder, highlighting the diverse impact on multiple aspects of daily life, such as work, education, relationships, independent living, and physical health (Murphy et al., 2016). Among these domains, employment is notably one of the impacted areas where many autistic individuals experience significant challenges.

### 1.1. Employment Challenges

Employment offers opportunities for autistic individuals to access social and financial benefits that support personal fulfillment, social integration, and growth in areas such as self-esteem and autonomy (Reichard et al., 2019). However, they have experienced a significantly lower employment rate than peers with other disabilities or without disabilities around the world (e.g., Office for National Statistics, 2021; Roux et al., 2021). Many autistic individuals face challenges in both obtaining and retaining employment. For example, they may have difficulty demonstrating behaviors and qualities that are typically valued in traditional job interviews, such as maintaining eye contact, staying on topic during conversations, recognizing facial cues, and listening without interruptions (Solomon, 2020). Difficulties with time management and organization also present challenges for this population during the job application process, particularly for prioritizing application tasks and responding to interview invitations (Adreon & Durocher, 2007). Even after securing employment, autistic workers may continue to struggle with adapting to the workplace, including (a) building interpersonal connections with neurotypical coworkers, (b) managing assigned tasks, (c) adjusting to new work routines, and (d) navigating physical and sensory environments (Waisman-Nitzan et al., 2019).

### 1.2. Autism and Assistive Technology (AT)

AT refers to any device or system that enhances the functioning of individuals with disabilities, while AT services involve the support needed to select, acquire, and effectively use those technologies (Assistive Technology Act, 1998). A variety of ATs have been developed as complementary tools to traditional vocational rehabilitation services, aiming to support autistic individuals in addressing employment-related challenges (Zhou et al., 2025). For instance, virtual reality has been used to help autistic job seekers practice interview skills, as it offers a consistent, controlled, and customizable environment for their learning needs (Smith et al., 2014, 2020). In addition, ATs, such as reminder mobile applications and video modeling, have been implemented to support on-the-job performance for autistic workers (Allen et al., 2012; Gentry et al., 2015).

Employment-related ATs for autistic individuals include a wide range of tools and systems developed to address barriers throughout the employment process. Common AT categories consist of augmentative and alternative communication (AAC) devices and conversation aids, cognitive support technologies like scheduling software and task reminders, virtual reality and other immersive virtual environments, and sensory regulation tools, such as noise-canceling headphones (Wang & Jeon, 2024; Zhou et al., 2025). Research evidence indicates that these different types of ATs can improve both objective skill acquisition (e.g., job interview and vocational skills) and subjective psychological outcomes (e.g., confidence, self-efficacy), promoting greater independence and improved employment outcomes (e.g., Bennett et al., 2010; Bross et al., 2020; Kumazaki et al., 2020). Despite their substantial potential to improve workplace success for autistic adults, questions remain regarding the extent to which current AT solutions align with individual needs and real-world employment demands.

The development of employment-related ATs for autistic individuals appears to follow a trend that emphasizes skill training, particularly job interview skills, during the pre-employment stage (Zhou et al., 2025). Additionally, these ATs have primarily functioned as tools through which autistic individuals passively receive instruction from job coaches, rather than as technologies actively used or controlled by the individuals themselves. Although studies have reported positive outcomes, high abandonment rates have been commonly reported, often due to issues related to design, cost, accessibility, and stigma (Tsvetkova et al., 2024). Given these mixed findings, it remains unclear whether the AT development trends truly reflect the needs and preferences of autistic individuals across the employment process.

It is important to acknowledge that access to these ATs depends not only on their existence or functionality but also on funding sources and systemic incentives. While workplace accommodations are legally mandated in some countries (e.g., under the Americans with Disabilities Act in the United States), enforcement and implementation vary. Addressing these systemic and financial barriers is critical to ensuring that AT innovations are both accessible and equitably distributed.

### 1.3. Purposes

The development and implementation of effective ATs and related services should be informed by the real-world needs of autistic individuals. Without a comprehensive understanding of these needs, there is a risk that technological advancements may not translate properly into meaningful support for this population. Despite growing interest in AT as a tool for improving employment outcomes, little is known about how autistic individuals themselves perceive their AT needs in the context of employment. The present needs assessment study aims to better understand the employment-related AT needs of autistic individuals by addressing the following research question:

#### Research Question

What employment-related activities do autistic individuals view as important for AT support, and how do they experience using current AT services?

## 2. Methods

### 2.1. Procedure

This study was granted exempt status by the Institutional Review Board (IRB) at the first author’s institute prior to data collection. Participants were recruited through Prolific, an online crowdsourcing platform widely used in social science and related fields to recruit diverse participant samples (Peer et al., 2017; Turner et al., 2021). Compared to other platforms, Prolific offers research-focused services that support rapid, high-quality, and cost-effective recruitment of participants with diverse demographics (Douglas et al., 2023; Palan & Schitter, 2018).

Eligible participants were required to (a) have received an autism-related diagnosis, either in childhood or adulthood; (b) be 18 years of age or older; (c) reside in the United States; and (d) be able to read written English. Although diagnostic status was based on self-report, this approach is consistent with current practices in online autism research and has been recognized as a practical and inclusive strategy for engaging this underrepresented population in community-based research that prioritizes the perspectives of autistic individuals outside of clinical or institutional settings (Ahmed et al., 2020; Rubenstein & Furnier, 2021).

On Prolific, the platform’s built-in pre-screeners were used to filter for and identify a sample pool of eligible participants. The informed consent and full survey were distributed only to individuals within this pool. Eligible participants were provided with a link to access the full survey. To ensure a consistent understanding of AT, participants were presented with the following definition from the Assistive Technology Act of 1998 before responding to AT-related items: “*Any item, piece of equipment, or product system, whether acquired commercially, modified, or customized, that is used to increase, maintain, or improve the functional capabilities of individuals with disabilities*” (Assistive Technology Act, 1998). Upon completion, they received a six-digit code to enter on Prolific as proof of completion and to receive compensation of USD 6.00.

### 2.2. Participants

A total of 511 participants completed the full survey. After eligibility check and raw data cleaning, 10 responses were removed because they did not meet the eligibility criteria for autism-related diagnosis. The final sample that entered analysis included 501 eligible autistic adult participants. See Table 1 for detailed information about the demographic characteristics of the final sample.

### 2.3. Measures

#### 2.3.1. AT Use

The first part of the AT needs assessment was designed by the research team to collect general information about participants’ AT use. It consisted of 10 items presented in multiple-choice, multiple-selection, and open-ended formats. These questions gathered information on participants’ current and past AT use (e.g., “Do you use any ATs regularly in your daily life?”; “Have you stopped using any assistive technologies that you previously used?”), the duration of use (e.g., “How long have you been using AT?”; “How long did you use the ATs before you stopped using it?”), receipt of AT-related services (e.g., “Did you receive any professional services to help you get and use your assistive technologies?”), and satisfaction with currently used AT. Satisfaction was rated on a 5-point Likert-type scale ranging from 1 (“Very dissatisfied”) to 5 (“Very satisfied”), with higher scores indicating greater satisfaction. Participants also responded to open-ended inquiries to elaborate on their satisfaction ratings, explain why they stopped using certain ATs, and share additional reflections on their overall experience with AT for employment purposes.

#### 2.3.2. AT and AT Service Needs

The second part of the AT needs assessment was developed to assess AT and AT service needs of autistic adults and was adapted from the structure of the VRTAC-QE Needs Assessment (Bishop et al., 2022; Tansey et al., 2023). It consisted of 23 items, representing activities across four domains relevant to the employment process: (a) skill development (6 items), (b) job development and placement (4 items), (c) job tasks (7 items), and (d) job retention (6 items). First, participants rated the importance of ATs in performing each activity to help them achieve or maintain competitive integrated employment, using a 5-point Likert-type scale (1 = “Not important”, 2 = “Somewhat important”, 3 = “Neutral”, 4 = “Important”, 5 = “Very Important”), with higher scores indicating more importance. Following this, participants were asked to rate the level of need for professionals to improve AT services for each activity to help them achieve or maintain competitive integrated employment, using a 5-point Likert-type scale (1 = “None, no need”, 2 = “A little need”, 3 = “Some need”, 4 = “Moderate need”, 5 = “High need”), with higher scores indicating greater needs. Cronbach’s alphas for the importance ratings were 0.970 overall and ranged from 0.899 to 0.922 across domains, while for the improvement needs ratings, the overall alpha was 0.976 and ranged from 0.919 to 0.947 across domains.

A weighted needs score was calculated for each activity within its respective domain, reflecting both importance and improvement ratings. The calculation involved the following steps: (1) a total importance score for each domain was computed by summing the importance ratings of all activities within that domain; (2) a relative importance score for each activity was calculated by dividing the individual importance rating by the total importance score for the domain; (3) a weighted needs score for each activity was then calculated by multiplying the relative importance score by the corresponding need rating; and (4) a total AT and AT service need score was obtained for each domain by summing the weighted needs scores of all activities within that domain, ranging from 1 to 5. Higher scores indicated higher levels of AT and AT service needs. For interpretive purposes, the scores were categorized into five levels: no need (1.00–1.49), little need (1.50–2.49), some need (2.50–3.49), moderate need (3.50–4.49), and high need (4.50–5.00).

$$T_{d}=\sum_{i=1}^{n_{d}}\frac{I_{d,i}N_{d,i}}{\sum_{i=1}^{n_{d}}I_{d,i}}$$

In this equation, *I_d_*_,*i*_ refers to the importance rating for activity *i* in domain *d*, and *n_d_* refers to the number of activities assessed in domain *d*. *N_d_*_,*i*_ indicates the needs rating for activity *i* in domain *d*. *T_d_* is the total weighted needs for domain *d*.

### 2.4. Data Analysis

The current study used a convergent mixed-methods analysis, as this approach can enrich our understanding of autistic individuals’ subjective experiences of using ATs (Fetters et al., 2013). Quantitative data were analyzed using the Statistical Package for the Social Sciences (SPSS, version 29.0). For the second part of the needs assessment, missing cases per item ranged from 1% to 2% per item. Little’s MCAR test indicated that the data were missing completely at random (χ2[1569] = 1615.89, *p* = 0.20). Therefore, missing values were treated using the expectation-maximization (EM) approach (Schlomer et al., 2010).

Harman’s single-factor test was conducted to assess potential common method bias (Podsakoff et al., 2003). All 23 items measuring the importance of ATs and the level of needs for AT services were entered into an unrotated principal axis factor analysis. Four factors had eigenvalues greater than one, with the first factor accounting for 52.22% of the variance. Although slightly above the 50% threshold, no single factor accounted for the majority of the variance, and item loadings were spread across multiple factors, suggesting that common method bias was unlikely to be a serious concern (Podsakoff et al., 2003). In addition, subgroup analyses were conducted to examine whether AT needs differed by age, highest educational level, and employment status. Pearson correlations were used for continuous variables (e.g., age), and one-way ANOVAs or non-parametric equivalents (Kruskal–Wallis tests) were used for categorical variables (e.g., education level, employment status).

Qualitative data from three open-ended questions, which focused on satisfaction with current AT use, discontinuation of AT use, and additional comments, were analyzed using a thematic analysis approach. The analysis followed Braun and Clark’s (Braun & Clarke, 2022) six-step process: (1) familiarization with data, (2) generating initial codes, (3) searching for themes, (4) reviewing themes, (5) defining and naming themes, and (6) producing the final report. Each question was assigned to a separate researcher who independently reviewed and coded responses. To enhance trustworthiness and consistency, the lead author conducted a secondary review of all coded data and emerging themes. Discrepancies were resolved collaboratively to ensure that identified themes accurately reflected participants’ perspectives. Themes were reported separately for each question, with representative participant quotes included to illustrate key findings.

## 3. Results

### 3.1. General AT Use

Of the 501 participants, 63.8% (*n* = 319) reported regularly using ATs in their daily lives. Commonly reported ATs included both information technology (IT) and non-IT solutions, including noise-canceling headphones, reminder and organization mobile applications, speech recognition technology, and smart assistants such as Alexa or Siri. When asked about the duration of AT use, 34.9% (*n* = 175) indicated that they had never used AT, 17.0% (*n* = 85) reported using AT for less than two years, and 46.5% (*n* = 233) had used AT for more than two years.

Regarding professional support, across the full sample, 87.0% (*n* = 436) reported never having received any AT-related services. By comparison, 8.2% (*n* = 41) had received assistance in obtaining AT from professionals such as occupational therapists, medical providers, vocational rehabilitation counselors, and AT specialists. A total of 4.2% (*n* = 21) had received support in using AT from professionals such as therapists, medical providers, and technical support staff. Only 1.8% (*n* = 9) had received services both in obtaining and using AT. These figures include both currently employed participants and those who were unemployed, retired, or students at the time of the survey, and thus reflects general AT service access rather than workplace-specific AT provision.

### 3.2. AT and AT Service Needs Assessment

**Figure 1:**
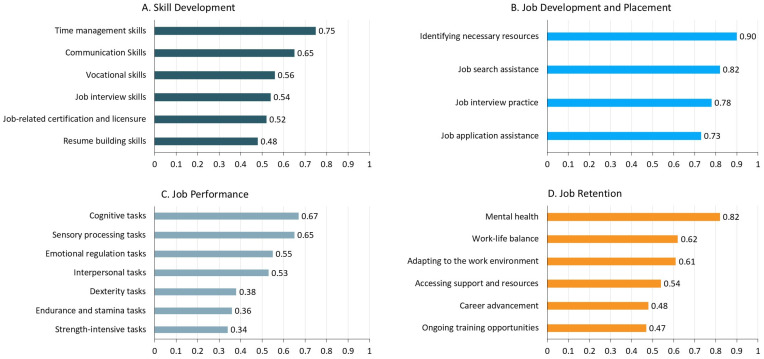
Weighted needs scores of activities across four domains.

Table 2 presents the descriptive statistics for the importance scores, the relative importance scores, the needs scores, the weighted needs scores, and the ranking of the weighted needs scores across the four domains (Figure 1).

In the skill development domain, “Time management skills”, “Communication skills”, and “Vocational skills” ranked highest in terms of weighted needs scores, while “Resume building skills” was rated lowest. The total weighted needs score for this domain was 3.51 (SD = 1.07), indicating a moderate level of AT and AT service needs among autistic adults.

For the job development and placement domain, the ranked weighted needs scores were as follows: (1) “Identifying necessary resources”, (2) “Job search assistance”, (3) “Job interview practice”, and (4) “Job application assistance (e.g., navigating application portal, preparing application materials).” The total weighted needs score for this domain was 3.22 (SD = 1.29), indicating some level of AT and AT service needs among autistic adults.

In the job performance domain, top-ranked activities included “Cognitive tasks (e.g., memory and learning, planning, task management)”, “Sensory processing tasks (e.g., noise management, vision acuity)”, and “Emotional regulation tasks (e.g., anger management, coping with change),” while “Strength-intensive tasks (e.g., lifting, carrying)” and “Endurance and stamina tasks (e.g., walking, standing)” ranked lower. The total weighted needs score was 3.48 (SD = 1.11), indicating some level of AT and AT service needs among autistic adults.

Finally, for the job retention domain, “Mental health”, “Work-life balance”, and “Adapting to the work environment” ranked highest in terms of weighted needs scores, while “Ongoing training opportunities” were rated lowest. The total weighted needs score was 3.53 (SD = 1.07), indicating a moderate level of AT and AT service needs among autistic adults.

In terms of subgroup comparisons, age was significantly associated with weighted needs scores across all four domains (r = 0.132 to 0.170, *p* < 0.05), with older participants reporting higher needs. No significant differences were found by highest educational level or employment status (all *p* > 0.05).

### 3.3. Satisfaction with Current AT Use

Participants were asked to elaborate on their satisfaction rating for their current use of AT (Table 3). A total of 426 participants responded, with a mean satisfaction rating of 4.06 (SD = 0.841) on a five-point Likert-type scale. Four primary themes emerged from the responses that appeared to influence participants’ satisfaction with AT: Access to Support and Training (*n* = 111), Accessibility and Availability of Technology (*n* = 49), Impact on Communication and Independence (*n* = 114), and Match Between Needs and Technology (*n* = 221). An additional 41 participants noted that they were not currently using any AT.

#### 3.3.1. Theme 1: Access to Support and Training

Participants highlighted the importance of professional support and guidance in selecting and using AT. Several expressed dissatisfaction with the lack of guidance, reporting that they were left to navigate AT use without training or a formal needs assessment. One participant noted, “*I need professional help to assess my needs for both my physical disabilities and Autism/ADHD. I have minimal tools currently.*” Another shared, “*I’ve never even been offered any kind of assistive technology. It could be helpful, but I wouldn’t know where to begin.*” These responses underscore the foundational role of individualized support in facilitating effective AT use.

#### 3.3.2. Theme 2: Accessibility and Availability of Technology

Participants described difficulties affording AT or accessing suitable options. As one explained, “I wish I could afford and access adaptive technology.” Another stated, “The things I can find that I can afford are very minimally helpful or designed for people with profound support needs and no one in the middle.” Some called for more discreet or readily accessible tools, such as one participant who said, “Using my phone as a magnifier is essential, but I wish I could access the same technology faster or less conspicuously.” This theme reflects barriers in affordability, availability, and design that restrict access to helpful tools.

#### 3.3.3. Theme 3: Impact on Communication and Independence

Participants consistently described AT as essential for communication and independence as well as sensory and emotional regulation. One shared, “*I have used AT to help me write ever since I was a kid… Voice to text has made the process of texting and making comments on mobile very easy.*” Others noted the value of specific tools in daily life: “*My smartwatch helps with reminders, Alexa assists with hands-free tasks, and ChatGPT offers quick information and support.*” Another participant emphasized broader benefits: “*There are a lot of various tools I use day-to-day to help with executive functioning and sensory-related issues that stem from being both autistic and with ADHD.*” These accounts emphasize how AT can empower autistic adults, enhance daily functioning, and support greater independence.

#### 3.3.4. Theme 4: Match Between Needs and Technology

Among those who expressed satisfaction, many still described their AT as only partially effective or poorly matched to their specific needs. “*It works for what I need it to for the most part. There’s likely a better technology that could help, but I’d likely not have the funds to acquire it,*” one participant noted. Another shared, “*What I use works well for me at the moment, but there are definitely other things that would probably help me out more.*” This theme reflects that satisfaction exists on a continuum, shaped by how well the AT aligns with individual needs.

### 3.4. Discontinuation of AT Use

Participants who reported discontinuing any AT use were asked to briefly explain their reasons. Of the 501 participants, 116 (23.2%) provided a response. The remaining 385 participants (76.8%) either left the answer blank or indicated “Not Applicable,” typically because they were current users or had no prior AT experience. Seventeen responses were excluded due to insufficient or unclear information. Two themes emerged from the valid responses: Issues Related to the AT (*n* = 57) and Growth and Desire for Independence (*n* = 42).

#### 3.4.1. Theme 1: Issues Related to the AT

The most frequently reported reason for discontinuing AT use was dissatisfaction with the device itself. Participants described past AT as unhelpful, frustrating, distracting, overly complicated, or uncomfortable. In some cases, updates made previously usable technologies less accessible. One participant explained, “*I stopped using the technology because it became too cumbersome, and the updates made it less user-friendly for my needs.*” These responses highlight how poor user interface, discomfort, and usability challenges can drive abandonment, underscoring the importance of intuitive, user-centered design in AT development.

#### 3.4.2. Theme 2: Growth and Desire for Independence

Some participants described discontinuing AT use after developing skills that reduced their reliance on these tools. For example, one participant shared, “*I used this to help me get better at mimicking normal social behavior and eye contact. I felt like I learned all I could and it improved my interactions for work and relationships.*” Another echoed this sentiment: “*It served its purpose for me… but now I feel empowered to manage my responsibilities independently, trusting myself to stay on track without relying on software.*” Others expressed a desire to build tolerance or physical strength without the aid of technology, such as one participant who noted, “*I figured I could develop my hand muscles more by typing even though I am a slow typer.*” These responses reflect how perceived personal growth, skill acquisition, and autonomy can motivate individuals to reduce or discontinue AT use.

### 3.5. Additional Comments

At the end of our survey, participants were invited to share any additional comments about their experience of AT use. A total of 168 participants responded. Three primary themes emerged from the responses: Little to No Experience with AT (*n* = 58), AT as a Game-Changer (*n* = 51), and Societal Misunderstanding of AT for Autistic Individuals (*n* = 34). A small number of comments (*n* = 5) pointed to a possible emerging theme related to the desire for AT that supports socio-emotional regulation in the workplace. Due to the nature of the survey design, follow-up was not possible to determine whether data saturation could be reached for potential emerging themes. The remaining responses were idiosyncratic or did not align with a broader pattern.

#### 3.5.1. Theme 1: Little to No Experience with AT

Many participants shared that they had limited or no prior experience using AT, but some expressed a desire to learn more about its potential benefits for employment pursuits. As one participant reflected, “*I really didn’t consider using assistive technologies, but I realize now how helpful it could be in my daily life. I wish that I had more resources to learn about AT, especially when I need them.*” Others highlighted a lack of education and outreach: “*I’ve been informed that I legally have access to AT but have never been educated about what types of AT would be helpful in specific roles nor have I been given any additional information about them.*” Some respondents noted they had concealed their disability in the workplace and were unfamiliar with available supports: “*I need to learn more about them. I have always masked my disability at my workplace.*” These responses suggest that many autistic adults lack exposure to or knowledge of AT, which limits their ability to access potentially beneficial AT services.

#### 3.5.2. Theme 2: AT as a Game-Changer

Many participants described AT as transformative for their work performance and overall productivity. AT was seen as enhancing organization, communication, and task management. One respondent stated, “*[A]ssistive technologies have been a game-changer for me in the workplace. They’ve helped me stay organized, communicate more effectively, and manage tasks that would otherwise be challenging. Having the right tools has made me feel more confident and productive in my job.*” Another added, “*They are life-saving on tasks and life.*” These responses underscored the positive impact and value of AT in facilitating workplace success.

#### 3.5.3. Theme 3: Societal Misunderstanding of AT for Autistic Individuals

Several participants described how societal attitudes, particularly in the workplace, created barriers to using AT. AT was described as “*still profoundly misunderstood in mainstream work environments,*” leading some to be denied access, experience prejudice, or avoid AT altogether due to fears of stigma or unintentional disclosure of their diagnosis. One participant explained, “*Some people seem to think that assistive technologies aren’t necessary, and that is very frustrating. It hurts to be called ‘dramatic’ or a ‘faker’ because I am trying to work for a living.*” Another expressed, “*I would be more likely to use them if I didn’t think they’d show my boss that I was clearly autistic.*” These responses highlight how misunderstanding and stigma from coworkers and supervisors may discourage AT adoption, even when it is needed.

## 4. Discussion

The purpose of the study was to explore autistic adults’ needs for AT and AT services across four domains of the employment process, as well as their subjective experiences with AT use. Comprehensive needs assessments of AT for autistic adults are scarce, and those that exist (e.g., Tsvetkova et al., 2024) have limited focus on employment. The findings offer important insights into autistic adults’ perceived AT needs and barriers across employment stages, highlighting discrepancies between their needs and current trends in AT development.

Among all four domains, job retention received the highest total weighted needs score, indicating that autistic adults have the greatest need for AT to support them in maintaining employment. Current AT development focuses primarily on the pre-employment stage, such as interview preparation (Zhou et al., 2025). Few ATs are specifically designed for job retention. However, findings from this study suggest that autistic adults prioritize AT and AT services for retaining employment over pre-employment support. The top-rated activities in this domain (i.e., “Mental health”, “Work-life balance”, and “Adapting to the work environment”) are all related to work adjustment, which involves helping individuals adapt to and find satisfaction in the workplace. Successful work adjustment not only enhances employee well-being but also benefits organizations through improved productivity and reduced turnover (Dahling & Librizzi, 2015). Despite this need, access to AT supporting work adjustment remains limited.

Consistent with the previous literature (Zhou et al., 2025), skill development also emerged as a critical area for AT use. Developing employability skills significantly improves employment outcomes for autistic individuals (Griffiths et al., 2024). However, existing ATs often focus narrowly on interview skills (Smith et al., 2020), while participants rated time management and communication as higher priorities. These skills are traditionally taught through coaching (Stark & Lindo, 2023). The interpersonal demands of coaching can create stress, making it difficult to actively engage in learning and leading to passive compliance with instruction. In addition, ATs for communication skills are often developed for educational settings and fail to capture the contextual demands of workplace interactions, such as disclosure (Klefbeck, 2023). These findings highlight the need for more context-specific AT interventions.

Although job performance ranked third, its weighted needs score was very close to those of the top two domains. Participants prioritized AT for supporting cognitive functioning, executive tasks, and interpersonal communication. To achieve positive employment outcomes, autistic adults must not only acquire necessary employability skills but also apply them effectively in real-world job settings. This highlights the need for ongoing support beyond initial training. AT has the potential to address gaps in staffing and provide continuous, individualized assistance (Klavina et al., 2024). Sensory processing tasks also ranked highly, consistent with the existing literature showing that autistic adults commonly experience challenges in managing environmental stimuli like noise and lighting (de Vries, 2021). However, as noted with job retention, there remains a lack of AT specifically designed to support autistic adults in the post-employment phase.

The job development and placement domain received the lowest weighted needs score with a relatively large difference compared to the other domains. This may reflect the availability of vocational rehabilitation services that already provide support in this area (Ipsen et al., 2019). Still, participants reported some level of need in identifying necessary resources and job search assistance. The saturation of the labor market and shifts in traditional work models have compelled autistic adults to adapt their job search strategies (Nicholas & Klag, 2020). The local labor market information provided by vocational rehabilitation services may no longer align with their evolving needs. The rise of digital platforms presents an opportunity for AT solutions that help users efficiently access diverse and up-to-date employment resources.

Subgroup analyses indicated that older participants reported higher AT and AT service needs across all four domains. One the one hand, this finding may reflect age-related decline in functional capacity, which can potentially increase the need for ATs in the workplace (Garçon et al., 2016; Wilson et al., 2009). On the other hand, older autistic individuals have accumulated life and work experiences and encountered a wide range of barriers throughout the employment process, providing them with greater awareness of where AT could be beneficial. Similar trends have been observed in broader disability populations, where older adults demonstrate higher perceived AT needs due to both functional changes and accumulated experiential knowledge (Agree & Freedman, 2011).

Qualitative results brought up some important issues on autistic adults’ experiences of AT. AT was described as essential for managing work-related tasks, supporting independence, and enhancing daily functioning. Participants who were satisfied with their AT highlighted its role in improving organization, time management, communication, and task completion, describing it as “a game changer.” These comments reflect the value of AT as a supportive aid and a facilitator of independence. For many, AT reduced reliance on human support and enabled them to meet workplace expectations more consistently. These findings support the view that AT should be framed as not just an accommodation but a tool for long-term personal and professional development (Beyer & Perry, 2013).

Despite these benefits, participants identified significant barriers limiting AT use. Financial barriers, including high costs and limited insurance coverage, were common among participants and consistent with the previous literature (O’Neill et al., 2020). It is important to notice that this study did not assess participants’ personal financial capability, so it is unclear whether the reported cost barriers reflect a real inability to afford AT or simply the perception that it is too expensive. This limitation should be considered when interpreting the high prevalence of reported financial barriers, as individual differences in disposable income or access to funding sources may influence these perceptions.

Another recurring issue was the lack of training support. Many reported receiving AT without any instruction or follow-up, leading to frustration and, in some cases, abandonment. Social stigma further discouraged AT use. Some participants avoided AT to prevent being judged by colleagues, while others feared it might make their autism visible. The barriers to AT are not solely technological; they are shaped by systemic, interpersonal, and environmental factors (Widehammar et al., 2019).

Another recurring issue was a mismatch between user needs and current AT service delivery. Many participants noted that they had limited information about AT or how to access it, while others expressed dissatisfaction with AT that was poorly suited to their individual needs and work environments. This mismatch suggests that current services often fail to inform autistic adults or tailor support to their needs, contributing to underuse of and disengagement from AT. Participants called for more customized and context-specific AT solutions. These findings underscore the importance of person-centered AT service models that incorporate user education, individualized professional guidance, and ongoing feedback mechanisms to promote sustained and meaningful use (Scherer, 2014).

Taken together, the quantitative and qualitative findings present a cohesive picture of autistic adults’ employment-related AT needs that extends the existing theoretical understanding of workplace support for neurodivergent populations. Quantitatively, higher weighted needs scores for job retention, skill development, and job performance underscore the centrality of sustained, context-specific supports throughout the employment cycle. Qualitatively, participants’ narratives highlighted how these needs manifest in daily work life, indicating that effective AT use is not merely a matter of device availability but also of alignment with personal goals, environmental demands, and social perceptions. The convergence of these data sources reinforces the notion that AT needs are shaped by both structural demands and subjective experiences, supporting the perspective of person–environment fit. By integrating both perspectives, the study advances a more nuanced framework in which AT development and service delivery must simultaneously address functional requirements and lived experiences to achieve positive long-term employment outcomes.

### 4.1. Implications

This study highlights the need to prioritize AT development for job retention and workplace adjustment rather than focusing solely on pre-employment stages. Expanding AT design and service provision for on-the-job support is critical. Participants expressed interest in AT for areas such as mental health, cognitive functioning, and sensory needs. These findings point to clear opportunities to develop and evaluate context-specific AT services, such as workplace-friendly noise-canceling devices and targeted stress management applications, to address post-employment challenges.

In addition, the higher AT needs reported by older participants suggest that AT development should also consider age-related functional changes and accumulated workplace experiences. Designing ATs that address both physical and cognitive changes, as well as the unique employment barriers faced by older autistic adults, may improve long-term employment success for this group.

AT abandonment rates remain high, often due to limited training and support (Tsvetkova et al., 2024). Vocational rehabilitation agencies must build structured onboarding and follow-up protocols into AT service delivery. Rehabilitation professionals do not need to master all AT devices but should be able to support their clients’ use effectively (Alshamrani et al., 2024). Developing basic AT literacy among rehabilitation professionals, offering mandatory consumer training, and establishing early-stage follow-up procedures could improve AT use and satisfaction.

To further address gaps in awareness, personalization, and equitable access, public-facing education initiatives around AT are needed. Involving autistic adults in the co-design of AT will help enhance usability and relevance (Newbutt et al., 2024). Future research must also evaluate ATs designed for independent use, along with other user-driven innovations, to ensure that development efforts reflect the real-world needs and lived experiences of autistic adults rather than perceived deficits.

Finally, addressing financial barriers is essential to ensuring equitable access to AT. Efforts should consider both the actual affordability of devices and services and users’ perceptions of cost, as either may prevent adoption. Incorporating funding assistance, insurance coverage options, or low-cost alternatives into AT service models could help reduce these barriers.

### 4.2. Limitations

Several limitations should be considered when interpreting and applying the findings of this study. First, participants were recruited through an online crowdsourcing platform, leading to a potential overrepresentation of individuals with high digital literacy, internet access, and interest in technology. This may limit the generalizability of the results. Second, AT needs were analyzed across the entire sample without stratifying by demographic or other characteristics. As a result, the variation in AT needs across different subgroups of autistic adults was not assessed. Third, the structure of the needs assessment was based on a general, conventional framework of employment. Some items may not fully capture domains or activities that reflect the unique and diverse experiences of autistic job seekers and employees. Lastly, the open-ended responses did not always specify the exact type of AT, and the examples provided may reflect a tendency for respondents to recall more familiar or readily accessible technologies, which could lead to an overrepresentation of IT-related AT in our findings.

## 5. Conclusions

This study provides important insights into AT needs and experiences of autistic adults across different employment stages. Findings highlight the need for more person-centered AT services that offer individualized guidance, responsive design, and context-specific support, particularly for job retention and workplace adjustment. Barriers such as limited access, lack of training, and stigma continue to hinder effective use. By aligning AT development with the lived experiences of autistic individuals, future research and practice can better support the employment outcomes and long-term autonomy of autistic adults in the workplace.

## Figures and Tables

**Table 1:** Descriptive statistics of sociodemographic information of the participants (N = 501).

Demographic Variables	*n* (%)	M (SD)
Age (years)	493 (98.4)	34.07 (10.96)
Gender		
Male	228 (45.5)	
Female	194 (38.7)	
Non-binary	52 (10.4)	
Transgender	19 (3.8)	
Other (e.g., “Genderfluid”, “Agender”)	6 (1.2)	
Prefer not to respond	2 (0.4)	
Race/Ethnicity		
White	330 (65.9)	
African American/Black	64 (12.8)	
Multiracial	61 (12.2)	
Hispanic or Latinx	21 (4.2)	
Asian/Pacific Islander	16 (3.2)	
Native American/American Indian	4 (0.8)	
Other (e.g., “Middle Eastern”, “Romani”)	3 (0.6)	
Prefer not to respond	2 (0.4)	
Other Disabilities		
Yes	284 (56.7)	
No	215 (42.9)	
Did not respond	2 (0.4)	
Age at diagnosis	500 (99.8)	18.75 (11.56)
Highest Educational Level		
Bachelor’s degree	155 (30.9)	
Some college, no degree	127 (25.3)	
Graduate or professional degree	96 (19.2)	
High school graduate (or equivalency/GED)	69 (13.8)	
Associate’s degree	45 (9.0)	
Less than high school	5 (1.0)	
Other (e.g., “Vocational, medical assistant”)	4 (0.8)	
Employment status		
Full-time employed only	236 (47.3)	
Unemployed	76 (15.2)	
Part-time employed only	69 (13.8)	
Self-employed only	40 (8.0)	
Student only	26 (5.2)	
Other (e.g., “disabled”, “homemaker”)	24 (4.8)	
Student with employment	23 (4.6)	
Retired	5 (1.0)	
Did not respond	2 (0.4)	
Primary Source of Financial Support		
Self	248 (49.5)	
Shared responsibility	100 (20.0)	
Another family member	86 (17.2)	
A spouse or partner	38 (7.6)	
A professional agency (e.g., SSI, SSDI)	18 (3.6)	
Other (e.g., “non-related roommates”)	9 (1.8)	
Did not respond	2 (0.4)	

**Table 2:** AT and AT service needs across four domains (N = 501).

	Importance M (SD)	Relative Importance M (SD)	Needs M (SD)	Weighted Needs M (SD)	Rank
A. Skill Development					
Time management skills	3.66 (1.40)	0.19 (0.07)	3.49 (1.34)	0.75 (0.45)	1
Communication Skills	3.46 (1.46)	0.18 (0.05)	3.63 (1.29)	0.65 (0.36)	2
Vocational skills	3.21 (1.37)	0.17 (0.05)	3.33 (1.34)	0.56 (0.29)	3
Job interview skills	3.14 (1.46)	0.16 (0.05)	3.50 (1.35)	0.54 (0.33)	4
Job-related certification and licensure	3.07 (1.44)	0.16 (0.05)	3.32 (1.37)	0.52 (0.33)	5
Resume building skills	2.96 (1.41)	0.15 (0.04)	3.25 (1.35)	0.48 (0.31)	6
Total needs				3.51 (1.07)	
B. Job Development and Placement					
Identifying necessary resources	3.24 (1.40)	0.27 (0.07)	3.53 (1.30)	0.90 (0.51)	1
Job search assistance	3.11 (1.47)	0.25 (0.06)	3.41 (1.36)	0.82 (0.47)	2
Job interview practice	3.03 (1.47)	0.24 (0.06)	3.45 (1.40)	0.78 (0.47)	3
Job application assistance (e.g., navigating application portal, preparing application materials)	2.94 (1.45)	0.24 (0.05)	3.32 (1.35)	0.73 (0.44)	4
Total needs				3.22 (1.29)	
C. Job Performance					
Cognitive tasks (e.g., memory and learning, planning, task management)	3.67 (1.38)	0.17 (0.06)	3.53 (1.39)	0.67 (0.41)	1
Sensory processing tasks (e.g., noise management, vision acuity)	3.62 (1.45)	0.17 (0.06)	3.60 (1.35)	0.65 (0.39)	2
Emotional regulation tasks (e.g., anger management, coping with change)	3.36 (1.45)	0.15 (0.04)	3.48 (1.38)	0.55 (0.33)	3
Interpersonal tasks (e.g., interacting with employers, co-workers, or customers)	3.30 (1.40)	0.15 (0.04)	3.57 (1.38)	0.53 (0.31)	4
Dexterity tasks (e.g., typing, using hand tools)	2.75 (1.52)	0.12 (0.04)	2.95 (1.44)	0.38 (0.28)	5
Endurance and stamina tasks (e.g., walking, standing)	2.66 (1.49)	0.12 (0.04)	2.87 (1.44)	0.36 (0.28)	6
Strength-intensive tasks (e.g., lifting, carrying)	2.59 (1.49)	0.12 (0.04)	2.79 (1.46)	0.34 (0.27)	7
Total needs				3.48 (1.11)	
D. Job Retention					
Mental health	3.87 (1.29)	0.20 (0.07)	3.84 (1.33)	0.82 (0.41)	1
Work-life balance	3.41 (1.41)	0.20 (0.11)	3.54 (1.33)	0.62 (0.29)	2
Adapting to the work environment	3.37 (1.41)	0.17 (0.05)	3.53 (1.33)	0.61 (0.35)	3
Accessing support and resources	3.17 (1.43)	0.16 (0.04)	3.58 (1.33)	0.54 (0.31)	4
Career advancement (e.g., promotion, networking opportunities)	3.01 (1.41)	0.15 (0.04)	3.42 (1.34)	0.48 (0.29)	5
Ongoing training opportunities	2.96 (1.42)	0.15 (0.04)	3.30 (1.34)	0.47 (0.29)	6
Total needs				3.53 (1.07)	

**Table 3:** Summary of key qualitative findings.

Section/Theme	Frequency (*n*)	Description	Illustrative Quotes
**Satisfaction with Current AT Use**			
Theme 1: Access to Support and Training	111	Importance of professional support and training; lack of guidance leads to dissatisfaction.	“I need professional help to assess my needs for both my physical disabilities and Autism/ADHD. I have minimal tools currently.”
Theme 2: Accessibility and Availability of Technology	49	Barriers due to affordability, design, or access.	“The things I can find that I can afford are very minimally helpful or designed for people with profound support needs and no one in the middle.”
Theme 3: Impact on Communication and Independence	114	AT supports communication, independence, sensory regulation, and executive functioning.	“My smartwatch helps with reminders, Alexa assists with hands-free tasks, and ChatGPT offers quick information and support.”
Theme 4: Match Between Needs and Technology	221	Satisfaction depends on alignment between AT features and individual needs.	“What I use works well for me at the moment, but there are definitely other things that would probably help me out more.”
Not currently using AT	41	Reported no AT use at present.	—
**Discontinuation of AT Use**			
Theme 1: Issues Related to the AT	57	AT abandoned due to poor usability, discomfort, or updates that reduced accessibility.	“I stopped using the technology because it became too cumbersome, and the updates made it less user-friendly for my needs.”
Theme 2: Growth and Desire for Independence	42	AT discontinued after skill development, personal growth, or desire for independence.	“It served its purpose for me… but now I feel empowered to manage my responsibilities independently.”
**Additional Comments**			
Theme 1: Little to No Experience with AT	58	Many lacked exposure or knowledge of AT but expressed interest in learning more.	“I really didn’t consider using assistive technologies, but I realize now how helpful it could be in my daily life.”
Theme 2: AT as a Game-Changer	51	AT described as transformative for productivity, organization, and confidence.	“[Assistive technologies] have been a game-changer for me in the workplace. They’ve helped me stay organized, communicate more effectively, and manage tasks that would otherwise be challenging.”
Theme 3: Societal Misunderstanding of AT	34	Misunderstanding, stigma, or fear of disclosure discourage AT use.	“It hurts to be called ‘dramatic’ or a ‘faker’ because I am trying to work for a living.”

## Data Availability

The data presented in this study are available on request from the corresponding author (the data are not publicly available due to privacy or ethical restrictions).
